# The Potential of MN_4_-GPs (M = Mn, Fe, Co, Ni, Cu, Mo) as Adsorbents for the Efficient Separation of CH_4_ from CO_2_ and H_2_S

**DOI:** 10.3390/ma18122907

**Published:** 2025-06-19

**Authors:** Shiqian Wei, Xinyu Tian, Zhen Rao, Chunxia Wang, Rui Tang, Ying He, Yu Luo, Qiang Fan, Weifeng Fan, Yu Hu

**Affiliations:** 1 School of New Energy Materials and Chemistry, Leshan Normal University, Leshan 614000, China; weishiqian@wx.lsun.edu.cn (S.W.); tianxinyu_lsnu@126.com (X.T.); raozhen_lsnu@163.com (Z.R.); wangchunxia0002@163.com (C.W.); tangrui_lsnu@163.com (R.T.); heying_lsnu@163.com (Y.H.); luoyu_lsnu@163.com (Y.L.); fq1893@foxmail.com (Q.F.); 13880579527@163.com (W.F.); 2 Leshan West Silicon Materials Photovoltaic and New Energy Industry Technology Research Institute, Leshan 614000, China; 3 Material Corrosion and Protection Key Laboratory of Sichuan Province, Zigong 643000, China

**Keywords:** transition metal porphyrin-like moieties embedded in graphene sheets, methane, carbon dioxide, hydrogen sulfide, density functional theory

## Abstract

Carbon dioxide (CO_2_) and hydrogen sulfide (H_2_S) as harmful gases are always associated with methane (CH_4_) in natural gas, biogas, and landfill gas. Given that chemisorption and physisorption are the key gas separation technologies in industry, selecting appropriate adsorbents is crucial to eliminate these harmful gases. The adsorption of CH_4_, CO_2_, and H_2_S has been studied based on the density functional theory (DFT) in this work to evaluate the feasibility of transition metal (M = Mn, Fe, Co, Ni, Cu, Mo) porphyrin-like moieties embedded in graphene sheets (MN_4_-GPs) as adsorbents. It was found that the interactions between gas molecules and MN_4_-GPs (M = Mn, Fe, Co, Ni, Cu, Mo) are different. The weaker interactions between CH_4_ and MN_4_-GPs (M = Co, Ni, Cu, Mo) than those between CO_2_ and MN_4_-GPs or between H_2_S and MN_4_-GPs are beneficial to the separation of CH_4_ from CO_2_ and H_2_S. The maximum difference in the interactions between gas molecules and MoN_4_-GPs means that MoN_4_-GPs have the greatest potential to become adsorbents. The different interfacial interactions are related to the amount of charge transfer, which could promote the formation of bonds between gas molecules and MN_4_-GPs to effectively enhance the interfacial interactions.

## 1. Introduction

Methane (CH_4_), a high-energy-intensity fuel and an important industrial chemical, can be obtained from natural gas, biogas, and landfill gas [1,2,3]. However, corresponding treatments have to be implemented to separate CH_4_ from harmful gases. For example, besides CH_4_ as the dominant component accounting for the 55~65% gas volume, biogas has 30~40% carbon dioxide (CO_2_) and trace water as well as hydrogen sulfide (H_2_S) and organic acid [3]. Among them, H_2_S as a toxic acidic gas is harmful to human health and has different adverse effects such as cognitive and motor impairment as well as olfactory fatigue or anosmia, which could occur at concentrations within 20 to 500 parts per million (ppm), while concentrations beyond 600 parts per million (ppm) carry the risk of causing death [4]. Moreover, the presence of H_2_S is responsible for the corrosion of industry facilities, especially pipelines, resulting in a great waste of materials and a significant safety hazard during production [5]. CO_2_ as an acid gas also needs to be eliminated given its corrosive property [6,7] and negative effect on calorific values [8,9]. Simultaneously, CO_2_ and H_2_S as problematic components also exist in natural gas and landfill gas [10]. Therefore, the effective separation of CH_4_ from CO_2_ and H_2_S is essential to avoid pipeline corrosion and to satisfy calorific value standards in practical applications [11].

Chemisorption and physisorption have become the key gas separation technologies in industry [12]. Zeolites, active alumina, activated carbon, silica gels, and molecular sieves as commercial adsorbents are commonly used for gas separation [13,14,15,16,17]. With higher demands and more complex application environments, a large number of new materials have emerged. Graphene, one of the most promising materials, exhibits striking physical and chemical properties due to its unique structure composed of sp^2^-hybridized carbon atoms [18,19,20,21,22,23,24]. The tunable structure and correspondingly changed properties confer graphene with a potential to effectively and selectively adsorb target gases. It has been demonstrated that the introduction of nonmetal and/or metal atoms is a feasible way to tune adsorbate–adsorbent interactions and realize gas separations [25,26,27,28]. Recently, transition metal porphyrin-like moieties embedded in graphene sheets (MN_4_-GPs) are frequently being studied as catalysts for oxygen reduction reaction, oxygen evolution reaction, hydrogen evolution reaction, and CO_2_ reduction reaction, among others [29]. In addition, MN_4_-GPs can be used as gas sensors with a high selectivity and sensitivity [30]. It is indicated that the interactions between gas molecules and MN_4_-GPs are tunable. Therefore, MN_4_-GPs as adsorbents have the potential to separate CH_4_, CO_2_, and H_2_S by their different interactions with the adsorbate.

Density functional theory (DFT) studies have been proved to be a reliable method to predict the properties of materials. Moreover, DFT studies can provide insights at the atomic and electronic levels. The adsorption behaviors of CH_4_, CO_2_, and H_2_S on pristine or modified graphene have been reported based on DFT calculations. Gao et al. [31] investigated the adsorption behaviors of CH_4_ and H_2_S and successfully improved the adsorption capacity of graphene by doping with Ni. Gui et al. [32] studied CH_4_, CO, and C_2_H_2_ adsorption on pristine graphene and Mn-doped graphene and found that C_2_H_2_ and CO adsorption can be enhanced after Mn doping, while CH_4_ adsorption is still weak. Besides transition metal doping, co-doping with nonmetal atoms, especially the formed transition metal porphyrin-like moieties, has also shown potential in effectively promoting tunable interactions between gas molecules and graphene. There are few works focusing on the adsorption of CH_4_, CO_2_, and H_2_S on MN_4_-GPs. Nosheen et al. [33] found that iron porphyrin-like moieties embedded in graphene sheets (FeN_4_-GPs) could adsorb CO, NO, and NO_2_ with stronger interactions than CO_2_, H_2_S, NH_3_, and SO_2_. The work of Chen et al. [34] revealed the different adsorption properties of MN_4_-GPs (M = Fe, Co, Ni, Cu, Ru, Rh, Pd, Ag, Ir, Pt, and Au) for CH_4_. However, to the best of our knowledge, the adsorption behaviors of CH_4_, CO_2_, and H_2_S have not been simultaneously compared to those of MN_4_-GPs. This is not conducive to effectively designing a MN_4_-GP structure to separate CH_4_ from CO_2_ and H_2_S. Thus, a better understanding of adsorbate–adsorbent interactions is essential to evaluate the feasibility of MN_4_-GPs as adsorbents.

The adsorption mechanisms of CH_4_, CO_2_, and H_2_S on MN_4_-GPs were studied in this work based on DFT calculations. As for the MN_4_-GPs, common transition metals at low prices, such as Mn, Fe, Co, Ni, Cu, and Mo, were taken into account. It is expected that this work could gain an insight into the interactions between gas molecules (CH_4_, CO_2_, H_2_S) and MN_4_-GPs (M = Mn, Fe, Co, Ni, Cu, Mo) and furthermore provide ideas to effectively design selective adsorbents for the achievement of CH_4_, CO_2_, and H_2_S separation.

## 2. Calculation Methods

Vienna ab initio simulation package (VASP v5.3) is frequently chosen to implement DFT calculations, and the reliability of its calculation results is widely recognized [35,36,37,38]. Thus, all DFT calculations within this work were performed using VASP. Core–electron interactions were described with the projector augmented wave (PAW) method [39]. Exchange–correlation energies were calculated by the Perdew, Burke, and Ernzerhof (PBE) method based on generalized gradient approximation (GGA) [40]. Spin polarization and dipole correction were employed to eliminate spurious interactions in periodic boundary calculations. Owing to the long-range van der Waals (vdW) interactions between gas molecules and MN_4_-GPs, the dispersion force-corrected DFT (DFT-D) method introduced by Grimme was employed [41]. Cut-off energies of plane-wave bases were set as 450 eV. The k-point meshes of 4 × 4 × 1 were used for all the calculations in this work. The convergence thresholds of energies and forces were 1 × 10^−4^ eV and 0.02 eV/Å, respectively. In order to ensure the reliability of the calculation results, convergence tests of cut-off energies, k-point mesh, force, and energy thresholds were implemented, as shown in the supporting information. The energy values remained stable at approximately −464.1 eV when the cut-off energy was set above 450 eV (Figure S1). This implied that a cut-off energy of 450 eV is sufficient for the calculations, and this setting, which has been used in other works [42], also demonstrated good performance. The energy values were consistently stable with a k-point mesh of 4 × 4 × 1, an energy convergence threshold of 1 × 10^−4^ eV, and a force convergence threshold of 0.02 eV/Å (Figures S2–S4). Therefore, the calculation parameter settings were validated as appropriate.

**Figure 1:**
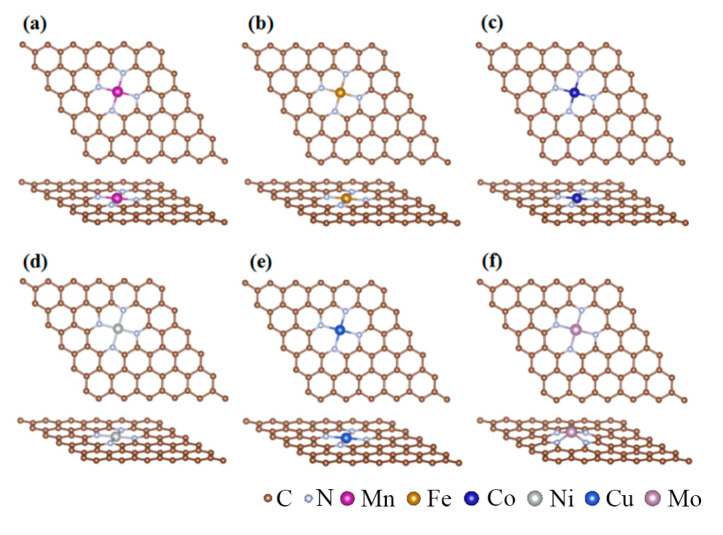
The structure of MN_4_-GPs: (**a**) MnN_4_-GPs; (**b**) FeN_4_-GPs; (**c**) CoN_4_-GPs; (**d**) NiN_4_-GPs; (**e**) CuN_4_-GPs; (**f**) MoN_4_-GPs.

The structures of MN_4_-GPs (M = Mn, Fe, Co, Ni, Cu, Mo) were constructed by loading transition metal single atoms coordinated with four nitrogen atoms on the (5 × 5) graphene supercells (*a* = *b* = 12.30 Å, *c* = 15.00 Å), as displayed in Figure 1. To evaluate the stability of MN_4_-GPs, the binding energies (*E*_b_) and the cohesive energies (*E*_c_) of transition metal single atoms (Mn, Fe, Co, Ni, Cu, Mo) loaded on nitrogen-doped graphene should be calculated.

*E*_b_ [43] can be obtained using the following equation:

*E*_b_ = *E*_MN4-GPs_ − *E*_M_ − *E*_N4-GPs_ (1)

where *E*_MN4-GPS_ is the total energy of different MN_4_-GPs, *E*_M_ is the energy of an isolated transition metal single atom, and *E*_N4-GPS_ is the energy of MN_4_-GPs without loading transition metal single atoms.

*E*_c_ [44] can be calculated using the following equation:

*E*_c_ = (*E*_bulk_ − *n* × *E*_M_)/*n* (2)

where *E*_bulk_ is the energy of the transition metal bulk phase, *n* is the number of atoms contained in the bulk phase, and *E*_M_ is the energy of an isolated transition metal single atom.

Different adsorption sites and orientations were considered to construct adsorption structures of CH_4_, CO_2_, and H_2_S on MN_4_-GPs. A total of 48 configurations were designed to study the adsorption behaviors of these molecules on MN_4_-GPs (M = Mn, Fe, Co, Ni, Cu, Mo). As shown in Figures S5–S10, CH_4_ molecules were adsorbed in three orientations: three hydrogen atoms were downward (labeled as (CH_4_)_3H-down_), three hydrogen atoms were upward (labeled as (CH_4_)_3H-up_), and two hydrogen atoms were downward (labeled as (CH_4_)_2H-down_). For CO_2_ molecules parallel to MN_4_-GPs, two adsorption structures were studied: an oxygen atom was adsorbed on the transition metal center (labeled as (CO_2_)_O-parallel_), and a carbon atom was adsorbed on the metal center (labeled as (CO_2_)_C-parallel_) (Figures S11–S16). In addition, the vertical adsorption structures of CO_2_ (labeled as (CO_2_)_O-vertical_) were also studied. For H_2_S molecules, both parallel and vertical adsorption structures were constructed on MN_4_-GPs, as shown in Figures S17–S22 (labeled as (H_2_S)_parallel_ and (H_2_S)_vertical_, respectively).

The thermodynamically stability of the adsorption structures can be evaluated by adsorption energies E_ads_ [45]:

*E*_ads_ = *E*_total_ − *E*_substrate_ − *E*_gas_ (3)

where *E*_total_ is the total energy of the MN_4_-GPs system with adsorbed gas molecules, *E*_substrate_ is the energy of the pristine MN_4_-GPs substrate, and *E*_gas_ is the energy of isolated gas molecules in the vacuum.

## 3. Results and Discussion

### 3.1. The Thermodynamic Stability of MN_4_-GPs

**Figure 2:**
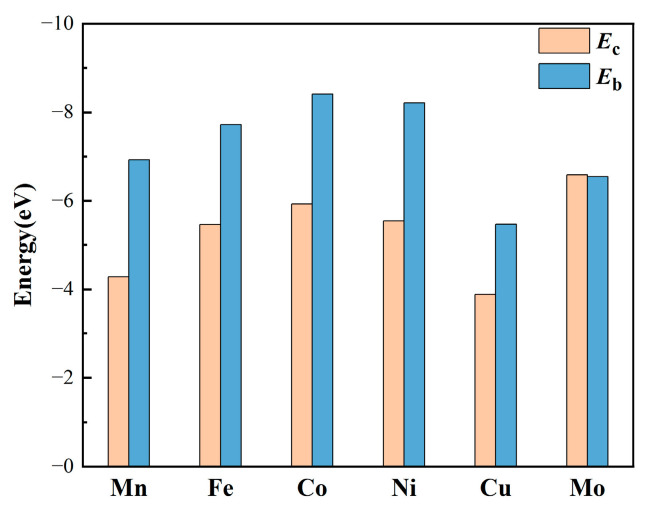
The binding energies *E*_b_ and cohesive energies *E*_c_ of transition metal single atoms in MN_4_-GPs (M = Mn, Fe, Co, Ni, Cu, Mo).

First of all, the thermodynamic stability of MN_4_-GPs (M = Mn, Fe, Co, Ni, Cu, Mo) should be taken into account. The binding energies (*E*_b_) and the cohesive energies (*E*_c_) are key data to evaluate the thermodynamic stability of MN_4_-GPs. The negative value of *E*_b_ means that the transition metal single atoms could be stably embedded in the graphene sheets as porphyrin-like moieties. According to Equation 1, the *E*_b_ values of MN_4_-GPs (M = Mn, Fe, Co, Ni, Cu, Mo) are −6.93, −7.72, −8.41, −8.22, −5.46, and −6.55 eV, respectively. Compared to the binding energy, a more negative cohesive energy indicates a greater tendency for transition metal atoms to agglomerate. Namely, for stable MN_4_-GPs, the binding energy must be more negative than the cohesive energy. According to Equation 2, the *E*_c_ values of Mn, Fe, Co, Ni, Cu, and Mo atoms are −4.28, −5.46, −5.93, −5.55, −3.88, and −6.58 eV, respectively. To facilitate the comparison of *E*_b_ and *E*_c_ values, the results have been compiled in Figure 2. It is evident that the binding energies of Mn, Fe, Co, Ni, and Cu single atoms are more negative than their cohesive energies, while the cohesive energy and binding energy of Mo single atom are comparable. Thus, transition metal single atoms prefer to coordinate with nitrogen atoms to form porphyrin-like moieties embedded in graphene sheets. In previous experimental studies [46,47,48,49], these transition metal single atoms have been successfully anchored on nitrogen-doped graphene. It is indicated that Mn, Fe, Co, Ni, Cu, and Mo atoms can be stably embedded in the graphene sheets. Therefore, the thermodynamically stable MN_4_-GPs (M = Mn, Fe, Co, Ni, Cu, Mo) have the potential to be used as adsorbents.

### 3.2. The Adsorption of CH_4_, CO_2_, and H_2_S on MN_4_-GPs

**Figure 3:**
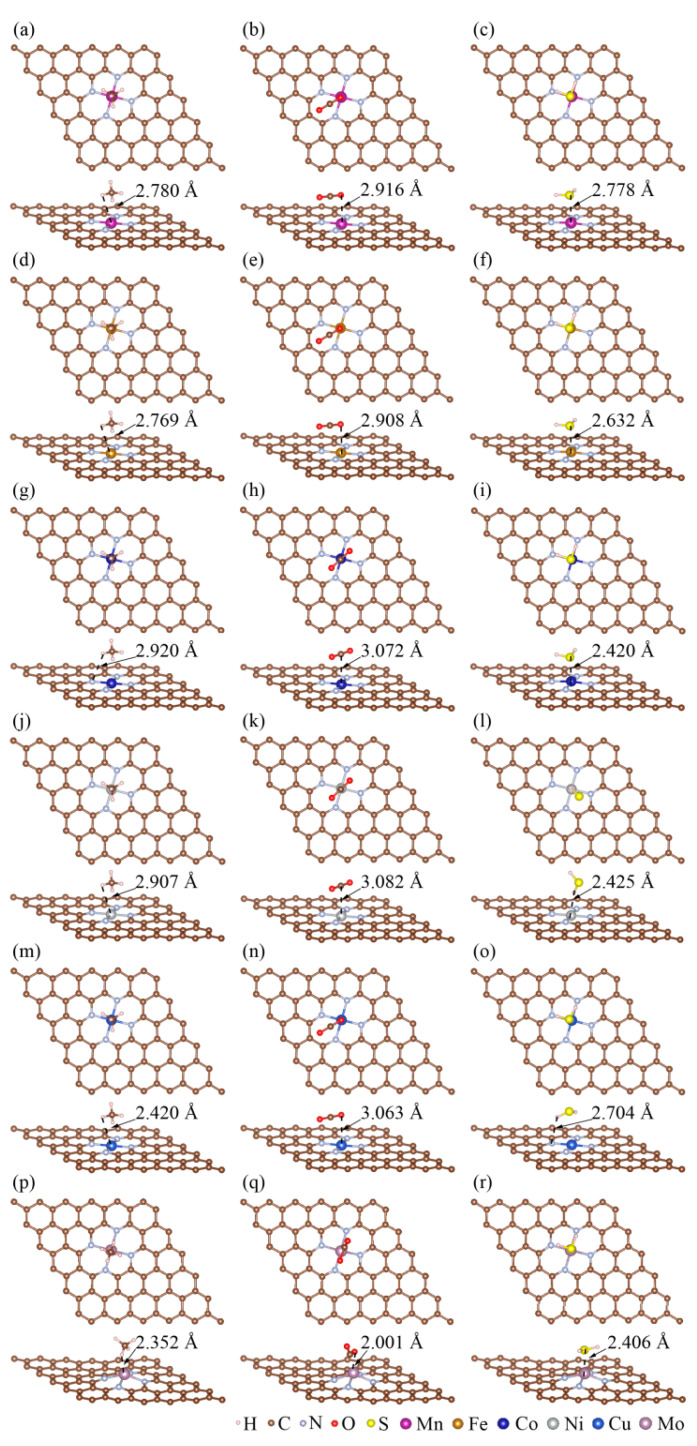
The most thermodynamically stable adsorption structures of CH_4_, CO_2_, and H_2_S on (**a**–**c**) MnN_4_-GPs, (**d**–**f**) FeN_4_-GPs, (**g**–**i**) CoN_4_-GPs, (**j**–**l**) NiN_4_-GPs, (**m**–**o**) CuN_4_-GPs, and (**p**–**r**) MoN_4_-GPs, respectively.

To identify the most thermodynamically stable adsorption structures of CH_4_, CO_2_, and H_2_S, various adsorption sites and orientations were explored (details are provided in Section 2). The negative values of adsorption energies (as shown in Table S1) represent the feasibilities of gas molecules’ adsorption on MN_4_-GPs. The more negative adsorption energies are, the more thermodynamically stable adsorption structures become. For example, the adsorption energies of (CH_4_)_3H-down_, (CH_4_)_2H-down_, and (CH_4_)_1H-down_ on MnN_4_-GPs are −0.19, −0.11, and −0.18 eV, respectively. It is indicated that (CH_4_)_3H-down_ on MnN_4_-GPs is the most thermodynamically stable among these three structures. Namely, CH_4_ molecules preferentially adsorbed on MnN_4_-GPs with three hydrogen atoms oriented downward (as shown in Figure 3a). In addition, (CO_2_)_O-parallel_ and (H_2_S)_S-parallel_ are the most thermodynamically stable configurations for CO_2_ and H_2_S on MnN_4_-GPs because the corresponding adsorption energies are the most negative, −0.18 and −0.34 eV, respectively. This indicates that CO_2_ and H_2_S adsorbed preferentially at Mn sites with their oxygen and sulfur atoms parallel to the MnN_4_-GPs substrate, respectively (as shown in Figure 3b,c). Similarly, the most thermodynamically stable structures of CH_4_, CO_2_, and H_2_S on FeN_4_-GPs, CoN_4_-GPs, NiN_4_-GPs, CuN_4_-GPs, and MoN_4_-GPs are screened out, as shown in Figure 3. CH_4_ molecules adsorb more readily on MN_4_-GPs, with three hydrogen atoms oriented down. The most thermodynamically stable orientations of CO_2_ are parallel to MN_4_-GPs, although the adsorption sites are different. H_2_S molecules adsorb horizontally on MN_4_-GPs, except NiN_4_-GPs. In summary, gas molecules tend to utilize as many atoms as possible to bond with MN_4_-GPs (M = Mn, Fe, Co, Ni, Cu, Mo), thereby achieving stable adsorption. Previous studies have also demonstrated the importance of interactions between gas molecules and material surfaces for thermodynamic stability [50,51,52]. In addition, the adsorption energies of CH_4_, CO_2_, and H_2_S on MN_4_-GPs obtained from other studies have been collected and are shown in Table S1. The adsorption energies of CO_2_ and H_2_S on FeN_4_-GPs calculated by Nosheen et al. [33] were −0.154 and −0.371 eV. Close values (−0.184 and −0.380 eV, respectively) are obtained in this work. Our calculation results are also consistent with the adsorption energies of CO_2_ and CH_4_ on FeN_4_-GPs, CoN_4_-GPs, NiN_4_-GPs, and CuN_4_-GPs in previous work [34]. This confirms the reliability of our results and provides theoretical support to gain an insight into CH_4_, CO_2_, and H_2_S adsorption behaviors on MN_4_-GPs.

### 3.3. The Feasibility of MN_4_-GPs for Efficient Separation of CH_4_ from CO_2_ and H_2_S

**Figure 4:**
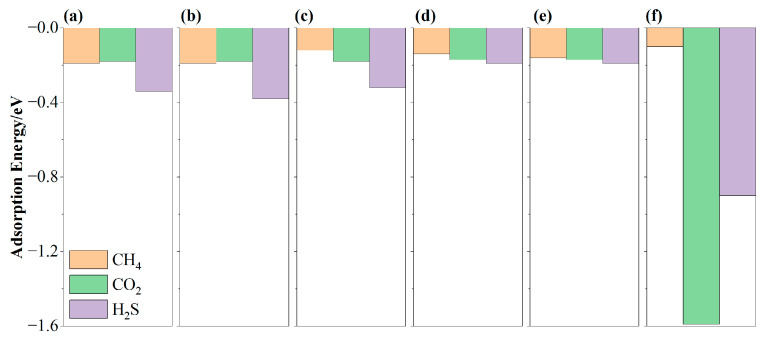
The adsorption energies of the optimal adsorption configurations of gas molecules on MN_4_-GPs: (**a**) MnN_4_-GPs, (**b**) FeN_4_-GPs, (**c**) CoN_4_-GPs, (**d**) NiN_4_-GPs, (**e**) CuN_4_-GPs, and (**f**) MoN_4_-GPs.

The negative adsorption energies of CH_4_, CO_2_, and H_2_S indicate that gas molecules can be stably adsorbed on MN_4_-GPs, while their values are different, as shown in Table S1. Adsorption energies are important references to evaluate the strengths of interactions. More negative adsorption energies imply stronger interactions. Thus, the varying interactions between gas molecules and MN_4_-GPs can be utilized to separate CH_4_ from CO_2_ and H_2_S. To conveniently observe these differences, the adsorption energies of the optimal adsorption configurations of gas molecules have been complied and are shown in Figure 4.

As shown in Figure 4a, the adsorption energies of (CH_4_)_3H-down_, (CO_2_)_O-parallel_, and (H_2_S)_S-parallel_ on MnN_4_-GPs are −0.19, −0.18, and −0.34 eV, respectively. This indicates stronger interactions between H_2_S and MnN_4_-GPs, while interactions involving CH_4_ and CO_2_ are comparable. Consequently, H_2_S can be separated from CH_4_ and CO_2_ due to the stronger adsorption on MnN_4_-GPs, while separating CH_4_ and CO_2_ is challenging because of the negligible difference in interactions. Similarly, the separation of CH_4_ and CO_2_ on FeN_4_-GPs is difficult given their close adsorption energies (−0.19 vs. −0.18 eV, as shown in Figure 4b). As atomic numbers increase, the interactions between CH_4_ and MN_4_-GPs (M = Co, Ni, Cu, Mo) (*E*_ads_ = −0.16~−0.10 eV) become weaker than the interactions between CO_2_ and MN_4_-GPs (M = Co, Ni, Cu, Mo) (*E*_ads_ = −1.59~−0.17 eV). Simultaneously, the interactions between H_2_S and MN_4_-GPs (M = Co, Ni, Cu, Mo) keep strong (*E*_ads_ = −0.90~−0.19 eV). Thus, adsorption on MN_4_-GPs (M = Co, Ni, Cu, Mo) enables the separation of CH_4_ from CO_2_ and H_2_S.

In addition, the adsorption capacity of MN_4_-GPs (M = Co, Ni, Cu, Mo) can be determined by the adsorption energy differences in CH_4_, CO_2_, and H_2_S. As shown in Figure 4, the adsorption energies of CH_4_ on CoN_4_-GPs, NiN_4_-GPs, CuN_4_-GPs, and MoN_4_-GPs are −0.12 eV, −0.14 eV, −0.16 eV, and −0.10 eV, respectively. This reveals that MoN_4_-GPs have the weakest adsorption capacity for CH_4_. The adsorption energies of CO_2_ on CoN_4_-GPs, NiN_4_-GPs, CuN_4_-GPs, and MoN_4_-GPs are −0.19 eV, −0.17 eV, −0.17 eV, and −1.59 eV, respectively. This implies that MoN_4_-GPs have the strongest adsorption capacity for CO_2_. The adsorption energies of H_2_S on CoN_4_-GPs, NiN_4_-GPs, CuN_4_-GPs, and MoN_4_-GPs are −0.32 eV, −0.19 eV, −0.19 eV, and −0.90 eV, respectively. MoN_4_-GPs exhibit the strongest adsorption capacity for H_2_S. In summary, MoN_4_-GPs show the weakest adsorption capacity for CH_4_ molecules but the strongest adsorption capacity for CO_2_ and H_2_S molecules. Therefore, among MN_4_-GPs (M = Co, Ni, Cu, Mo), MoN_4_-GPs are the optimal adsorbent for separating CH_4_ from CO_2_ and H_2_S.

Previous calculational studies have investigated adsorbent feasibility for CH_4_/CO_2_/H_2_S separation. Braga et al. [53] used DFT calculations to screen transition metal-exchanged Y zeolites for selective H_2_S/COS/CO_2_ removal from natural gas. Sokhanvaran et al. [12] employed grand canonical Monte Carlo simulations, demonstrating metal–organic frameworks (MOFs) as effective H_2_S-selective adsorbents. Zhan et al. [54] combined grand canonical Monte Carlo (GCMC) and molecular dynamics (MD) simulations to investigate the adsorption behavior of CO_2_/CH_4_ and H_2_S/CH_4_ mixtures within quartz nanopores. Aksu et al. [17] implemented the multi-level computational screening of covalent organic frameworks (COFs) for the capture of H_2_S+CO_2_ from natural gas. No prior work has examined MN_4_-GPs as adsorbents for the efficient separation of CH_4_ from CO_2_ and H_2_S. Our calculations reveal the adsorption behaviors of CH_4_, CO_2_, and H_2_S on different MN_4_-GPs, demonstrating that MN_4_-GPs (M = Co, Ni, Cu, Mo), particularly MoN_4_-GPs, are promising selective adsorbents for removing H_2_S and CO_2_ from natural gas, biogas, and landfill gas.

### 3.4. The Adsorption Mechanisms of CH_4_, CO_2_, and H_2_S on MN_4_-GPs

**Figure 5:**
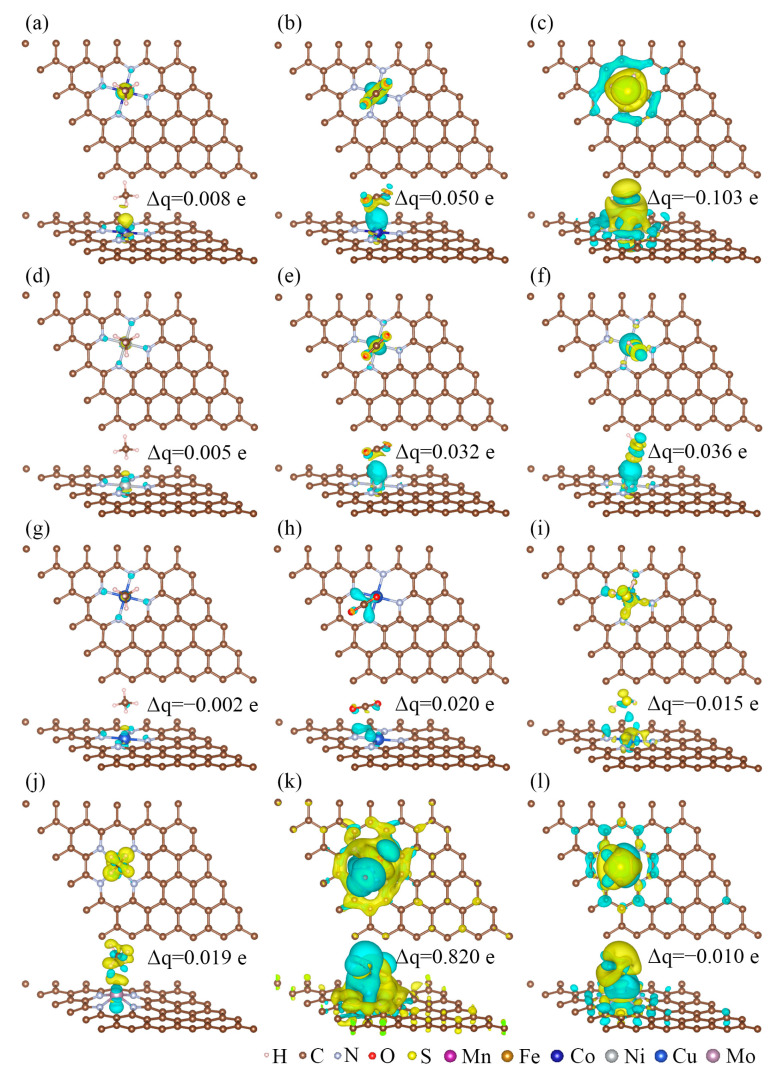
The difference charge density maps of CH_4_, CO_2_, and H_2_S on (**a**–**c**) CoN_4_-GPs, (**d**–**f**) NiN_4_-GPs, (**g**–**i**) CuN_4_-GPs, and (**j**–**l**) MoN_4_-GPs with the same isosurface value of 5 × 10^−4^ electrons/Å^3^, respectively.

The electronic properties of CH_4_, CO_2_, and H_2_S on MN_4_-GPs (M = Co, Ni, Cu, Mo) have been studied to gain an insight into their adsorption behaviors, which could explain the potential of MN_4_-GPs for efficiently separating CH_4_ from CO_2_ and H_2_S. According to the difference charge density maps, as shown in Figure 5, significant electron transfer occurs at the interfaces, where the yellow and blue regions represent electron accumulation and depletion, respectively. Thus, interactions between gas molecules and MN_4_-GPs (M = Co, Ni, Cu, Mo) are confirmed by interfacial charge redistribution. However, interaction strength varies due to differences in the spatial extent of charge redistribution. For example, the interfacial charge redistribution regions between CH_4_ and CoN_4_-GPs (Figure 5a) are smaller than those for CO_2_ (Figure 5b) or H_2_S (Figure 5c). Moreover, Bader charge analyses have been performed to quantify the amount of charge transfer Δ*q* (as labeled in Figure 5). There are 0.008 e electrons flowing from CoN_4_-GPs to CH_4_ (Figure 5a), 0.050 e electrons flowing from CoN_4_-GPs to CO_2_ (Figure 5b), and 0.103 e electrons flowing from H_2_S to CoN_4_-GPs (Figure 5c). Namely, compared with CH_4_ on CoN_4_-GPs, more electrons transfer at the interfaces of CO_2_ or H_2_S on CoN_4_-GPs. This trend is similar to that for other MN_4_-GPs (M = Ni, Cu, Mo). Therefore, the interactions between CO_2_ and MN_4_-GPs or between H_2_S and MN_4_-GPs are stronger than those between CH_4_ and MN_4_-GPs. As a result, the adsorption energies of CH_4_ are more positive than those of CO_2_ and H_2_S, as described in Section 3.3.

**Figure 6:**
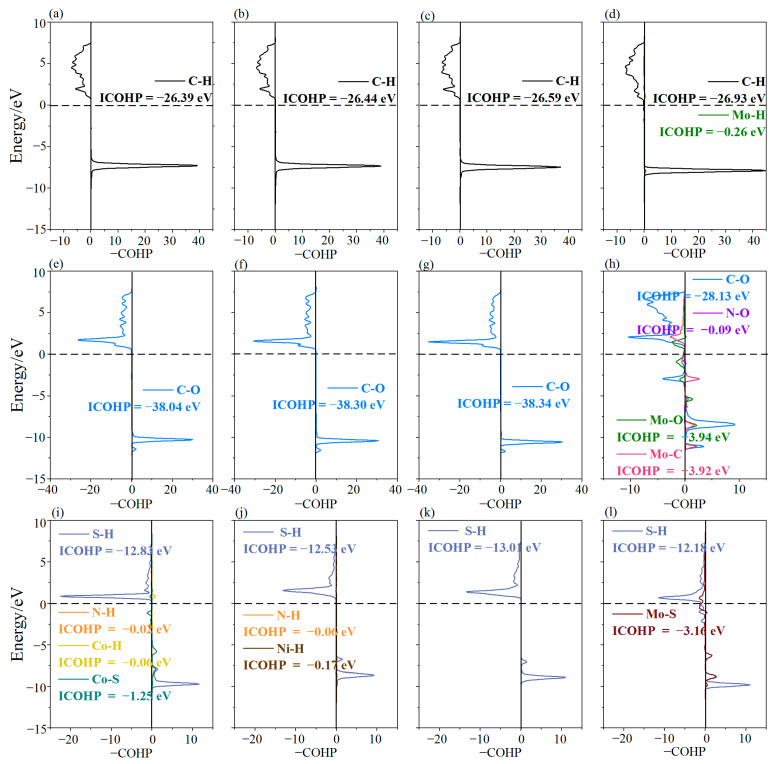
The COHP curves and ICOHP values of (**a**–**d**) CH_4_ on MN_4_-GPs (M = Co, Ni, Cu, Mo), (**e**–**h**) CO_2_ on MN_4_-GPs (M = Co, Ni, Cu, Mo), and (**i**–**l**) H_2_S on MN_4_-GPs (M = Co, Ni, Cu, Mo), respectively.

Crystal Orbital Hamilton Populations (COHPs) [55] are usually employed to analyze chemical bonds, while Integrated Crystal Orbital Hamilton Populations (ICOHPs), which are integral values of COHPs below Fermi levels, can quantify bonding electrons and evaluate bonding strength. More negative ICOHP values indicate stronger interactions. Thus, to further study the interactions between gas molecules and MN_4_-GPs (M = Co, Ni, Cu, Mo), COHP curves and ICOHP values are presented in Figure 6. For CH_4_ on MN_4_-GPs (M = Co, Ni, Cu), there are only COHP curves of C-H bonds (Figure 6a–c). Although COHP curves of Mo-H bonds are observed for CH_4_ on MoN_4_-GPs, those of C-H bonds show no significant changes, as shown in Figure 6d. In addition, the ICOHP values of C-H bonds in CH_4_ on MN_4_-GPs (M = Co, Ni, Cu, Mo) are −26.39, −26.44, −26.59, and −26.93 eV, respectively. Similar COHP curves and comparable ICOHP values indicate that minimal charge transfer (|Δ*q*| = 0.002~0.020) at the interface has a negligible impact on C-H bond strength, merely facilitating‌ weak adsorption interactions between CH_4_ and MN_4_-GPs (M = Co, Ni, Cu, Mo). The interactions between CO_2_ and MN_4_-GPs (M = Co, Ni, Cu) are comparable according to the similar COHP curves (Figure 6e–g) and close ICOHP values (−38.04, −38.30, and −38.34 eV). In contrast, the interaction between CO_2_ and MoN_4_-GPs strengthens significantly due to the formation of Mo-C (ICOHP = −3.92 eV), Mo-O (ICOHP = −3.94 eV), and N-O (ICOHP = −0.09 eV) bonds. This weakens C-O bonds in CO_2_, which can be verified by the appearance of an anti-bonding orbital peak below the Fermi energy level (Figure 6h) and a more positive ICOHP value (−28.13 eV). S–H bond strength is similarly modulated upon H_2_S adsorption on MN_4_-GPs (M = Co, Ni, Cu, Mo) (Figure 6i–l). For MoN_4_-GPs, the COHP curve of S-H bonds exhibits distinct changes, including a prominent ‌anti-bonding orbital peak below the Fermi energy level (Figure 6l). This is attributed to Mo-S bond formation (ICOHP = −3.16 eV). In conclusion, interfacial charge transfer critically drives bond formation between gas molecules and MN_4_-GPs, which plays an important role in enhancing their interactions. Therefore, among MN_4_-GPs (M = Co, Ni, Cu, Mo), MoN_4_-GPs exhibit the strongest adsorption capacity for CO_2_ and H_2_S molecules, which is consistent with the findings outlined in Section 3.3.

Physical adsorption is an alternative technology for the removal of H_2_S and CO_2_ from natural gas, biogas, and landfill gas, and the development of new/modified materials is an attractive research area in this regard [56]. Previous studies confirm that adsorbent selectivity can be tuned by introducing different transition metals [53,57]. Through DFT calculations, this work further reveals the essential reasons for atomic and electronic levels. Additionally‌, factors including gas molecular ratios, pressure, and temperature significantly influence adsorbent selectivity [58]. Future studies must quantify these parameters to establish a complete theoretical framework for designing MN_4_-GPs as adsorbents.

## 4. Conclusions

In this work, the adsorption of CH_4_, CO_2_, and H_2_S on MN_4_-GPs (M = Mn, Fe, Co, Ni, Cu, Mo) has been studied by constructing 48 adsorption configurations based on DFT calculations. The results reveal distinct adsorption orientations: CH_4_ molecules prefer a three-hydrogen-down geometry, while CO_2_ and H_2_S molecules adopt horizontal orientations. The adsorption energies of CH_4_ (−0.16~−0.10 eV) on MN_4_-GPs (M = Co, Ni, Cu, Mo) are more positive than those of CO_2_ (−1.59~−0.17 eV) and H_2_S (−0.90~−0.19 eV), indicating weaker interactions between CH_4_ and MN_4_-GPs. This difference facilitates separating CH_4_ from CO_2_ and H_2_S via MN_4_-GPs (M = Co, Ni, Cu, Mo). Gas–adsorbent interaction strength ‌scales linearly with charge transfer magnitude. Compared with CH_4_ (0.0023~0.019 e), more electrons are transferred during CO_2_ (0.020~0.82 e) and H_2_S (0.010~0.10 e) adsorption on MN_4_-GPs (M = Co, Ni, Cu, Mo). Moreover, on MoN_4_-GPs, the adsorption energy of CH_4_ (−0.10 eV) is the most positive, while the adsorption energies of CO_2_ (−1.59 eV) and H_2_S (−0.90 eV) are the most negative. Namely, MoN_4_-GPs exhibit the weakest adsorption for CH_4_ but the strongest for CO_2_ and H_2_S. The strong interactions of H_2_S and CO_2_ with MoN_4_-GPs are attributed to chemical bonding, specifically Mo-C and Mo-O bonds for CO_2_ and Mo-S bonds for H_2_S. Therefore, MoN_4_-GPs are the most promising adsorbent for the effective separation of CH_4_ from CO_2_ and H_2_S. It is expected that this work will provide a deep insight into the fundamental reasons for the different selectivity of MN_4_-GPs and offer a theoretical basis for designing effective adsorbents for the removal of H_2_S and CO_2_ from natural gas, biogas, and landfill gas.

## Data Availability

The original contributions presented in this study are included in the article/Supplementary Materials. Further inquiries can be directed to the corresponding author.

## Supplementary Materials

The following supporting information can be downloaded at: https://www.mdpi.com/article/10.3390/ma18122907/s1, Figure S1: The convergence test of cut-off energy; Figure S2: The convergence test of K-point mesh; Figure S3: The convergence test of force thresholds; Figure S4: The convergence test of energy thresholds; Figure S5: The structures of CH_4_ on MnN_4_-GPs; Figure S6: The structures of CH_4_ on FeN_4_-GPs; Figure S7: The structures of CH_4_ on CoN_4_-GPs; Figure S8: The structures of CH_4_ on NiN_4_-GPs; Figure S9: The structures of CH_4_ on CuN_4_-GPs; Figure S10: The structures of CH_4_ on MoN_4_-GPs; Figure S11: The structures of CO_2_ on MnN_4_-GPs; Figure S12: The structures of CO_2_ on FeN_4_-GPs; Figure S13: The structures of CO_2_ on CoN_4_-GPs; Figure S14: The structures of CO_2_ on NiN_4_-GPs; Figure S15: The structures of CO_2_ on CuN_4_-GPs; Figure S16: The structures of CO_2_ on MoN_4_-GPs; Figure S17: The structures of H_2_S on MnN_4_-GPs; Figure S18: The structures of H_2_S on FeN_4_-GPs; Figure S19: The structures of H_2_S on CoN_4_-GPs; Figure S20: The structures of H_2_S on NiN_4_-GPs; Figure S21: The structures of H_2_S on CuN_4_-GPs; Figure S22: The structures of H_2_S on MoN_4_-GPs; Table S1: The adsorption energies (eV) of CH_4_, CO_2_, and H_2_S on MN_4_-GPs (M = Mn, Fe, Co, Ni, Cu, Mo).
