# Supplementary material for: The Potential of MN4-GPs (M = Mn, Fe, Co, Ni, Cu, Mo) as Adsorbents for the Efficient Separation of CH4 from CO2 and H2S
Source: Materials (Basel). 2025 Jun 19;18(12):2907. doi: 10.3390/ma18122907 (PMC12195146; doi:10.3390/ma18122907)
Supplement: Supplementary file 1 [file materials-18-02907-s001.zip › materials-3663211-supplementary.pdf]

Supplementary Materials

The Potential of MN_4-GPs (M = Mn, Fe, Co, Ni, Cu, Mo) as Adsorbents for the Efficient Separation of CH_4 from CO_2 and H_2S

Shiqian Wei [1,2,3], Xinyu Tian [1], Zhen Rao [1], Chunxia Wang [1], Rui Tang [1], Ying He [1], Yu Luo [1], Qiang Fan [1,2], Weifeng Fan [1,2] and Yu Hu [1,2,*]

[1] School of New Energy Materials and Chemistry, Leshan Normal University, Leshan 614000, China; weishiqian@wx.lsun.edu.cn (S.W.); tianxinyu_lsnu@126.com (X.T.); raozhen_lsnu@163.com (Z.R.); wangchunxia0002@163.com (C.W.); tangrui_lsnu@163.com (R.T.); heying_lsnu@163.com (Y.H.); luoyu_lsnu@163.com (Y.L.); fq1893@foxmail.com (Q.F.); 13880579527@163.com (W.F.)

[2] Leshan West Silicon Materials Photovoltaic and New Energy Industry Technology Research Institute, Leshan 614000, China

[3] Material Corrosion and Protection Key Laboratory of Sichuan Province, Zigong 643000, China

* Correspondence: huyugucas@126.com

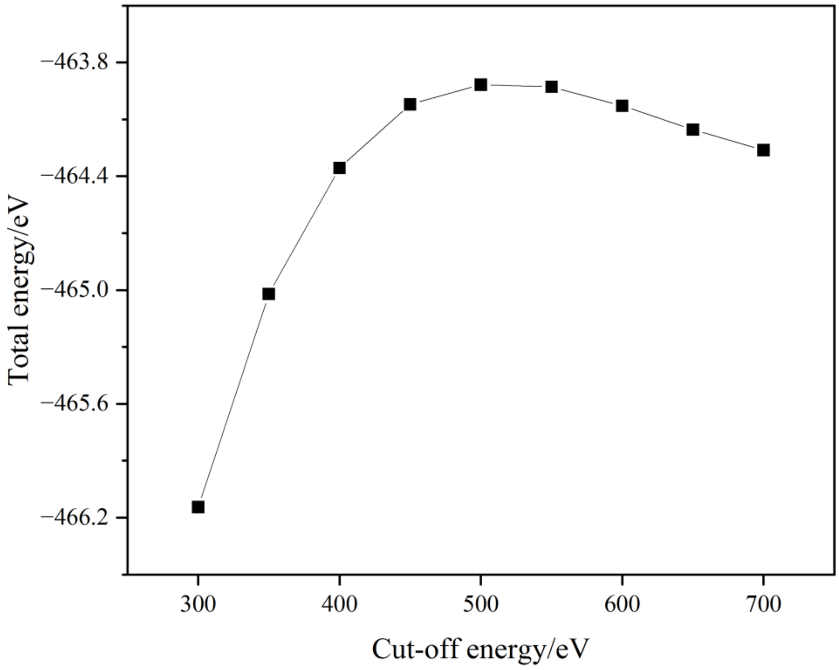

Figure S1. The convergence test of cut-off energy.

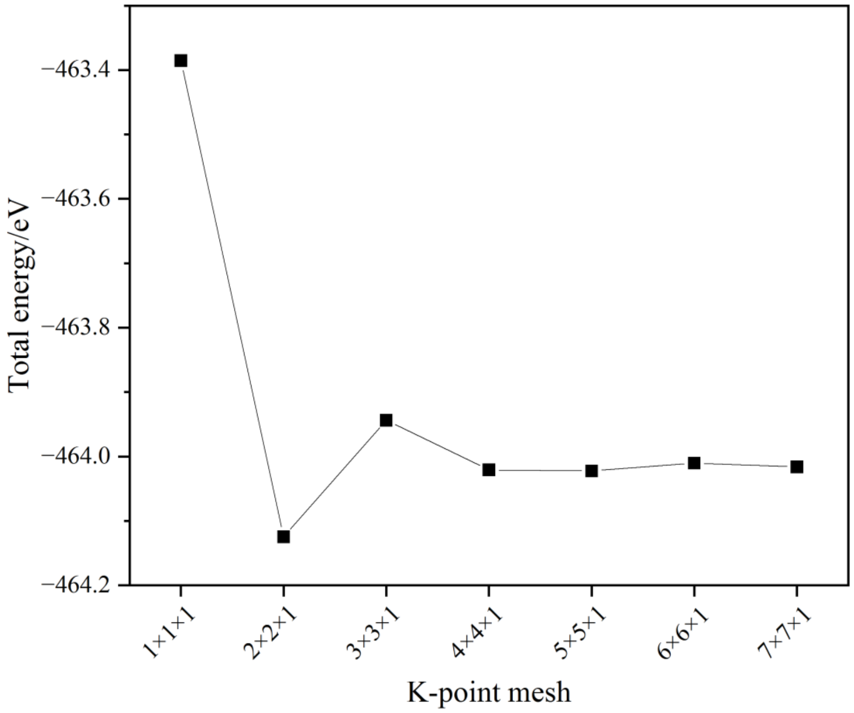

Figure S2. The convergence test of K-point mesh.

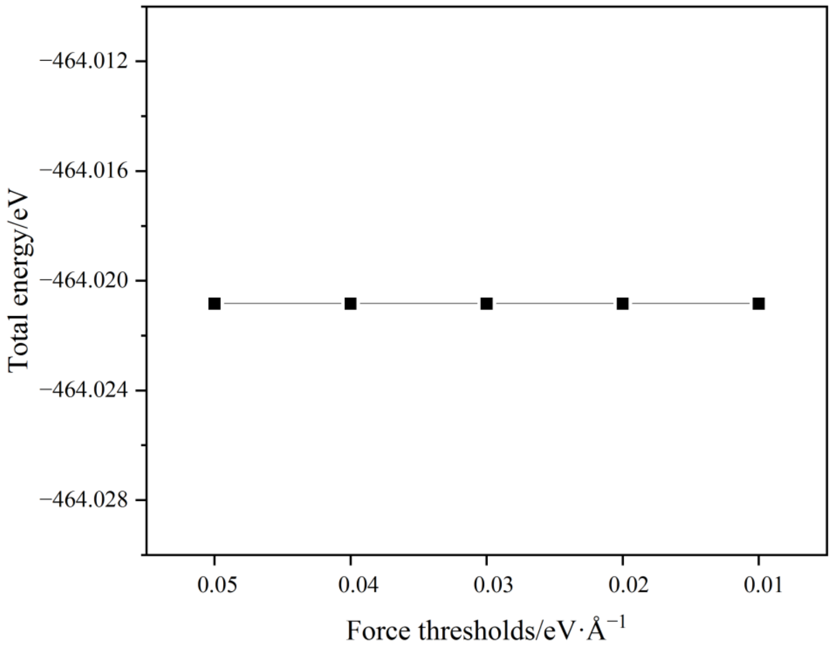

Figure S3. The convergence test of force thresholds.

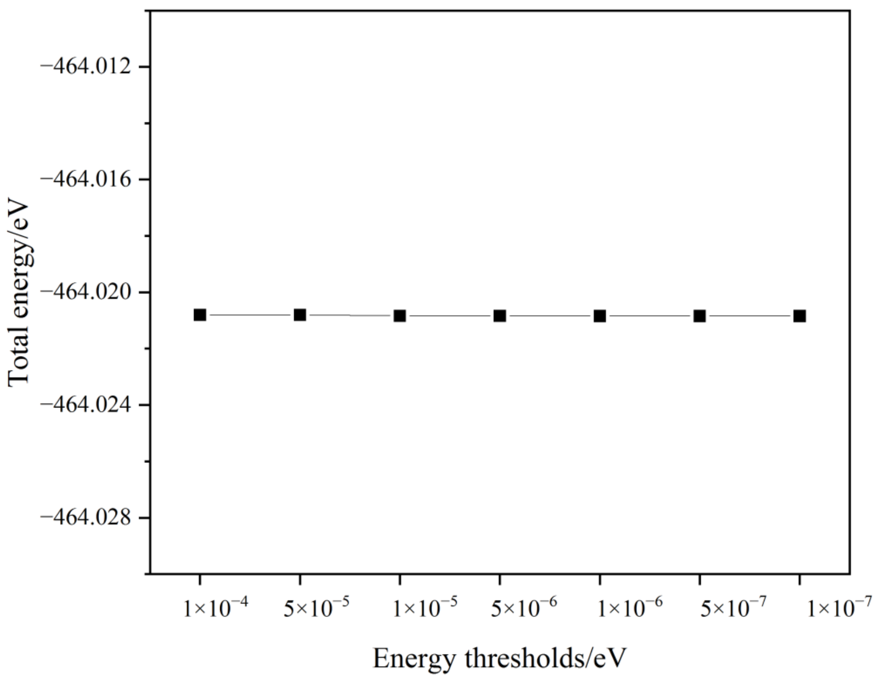

Figure S4. The convergence test of energy thresholds.

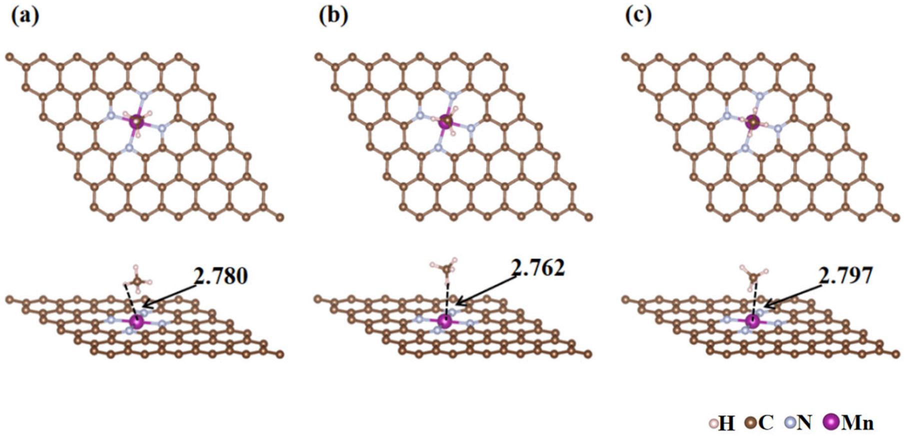

Figure S5. The structures of CH_4 on MnN_4-GPs: (a) $(CH_4)_{3H\text{-}down}$; (b) $(CH_4)_{3H\text{-}up}$; (c) $(CH_4)_{2H\text{-}down}$.

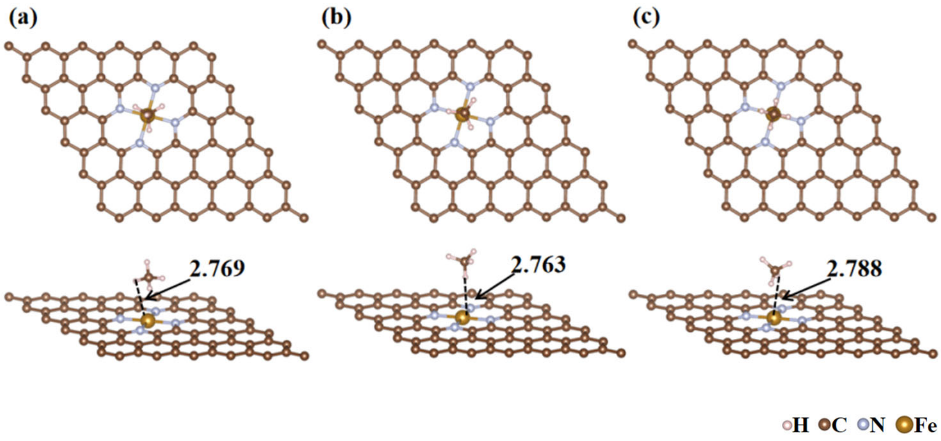

Figure S6. The structures of CH_4 on FeN_4-GPs: (a) $(CH_4)_{3H\text{-}down}$; (b) $(CH_4)_{3H\text{-}up}$; (c) $(CH_4)_{2H\text{-}down}$.

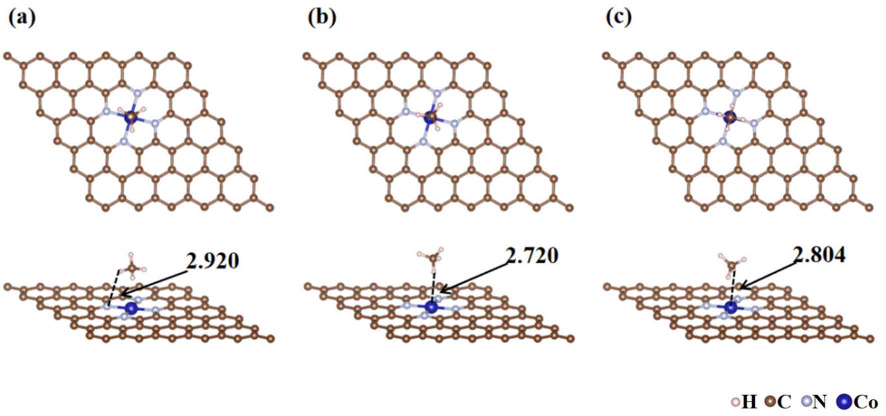

Figure S7. The structures of CH_4 on CoN_4-GPs: (a) $(CH_4)_{3H\text{-}down}$; (b) $(CH_4)_{3H\text{-}up}$; (c) $(CH_4)_{2H\text{-}down}$.

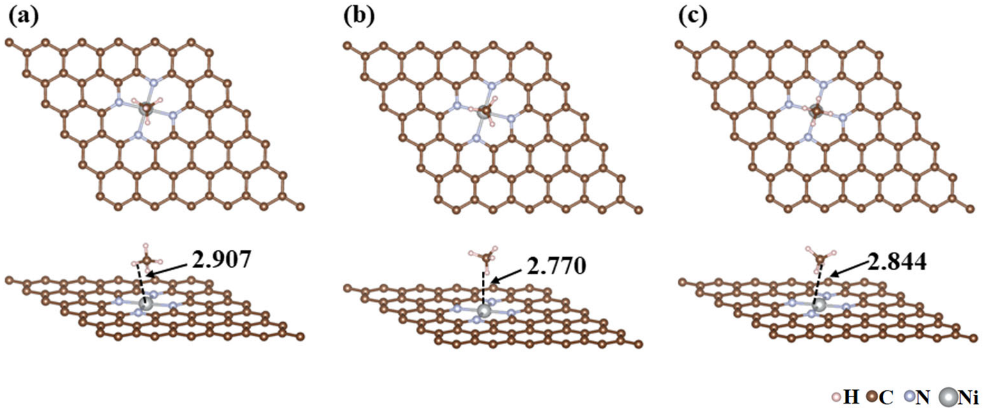

Figure S8. The structures of CH_4 on NiN_4-GPs: (a) $(CH_4)_{3H\text{-}down}$; (b) $(CH_4)_{3H\text{-}up}$; (c) $(CH_4)_{2H\text{-}down}$.

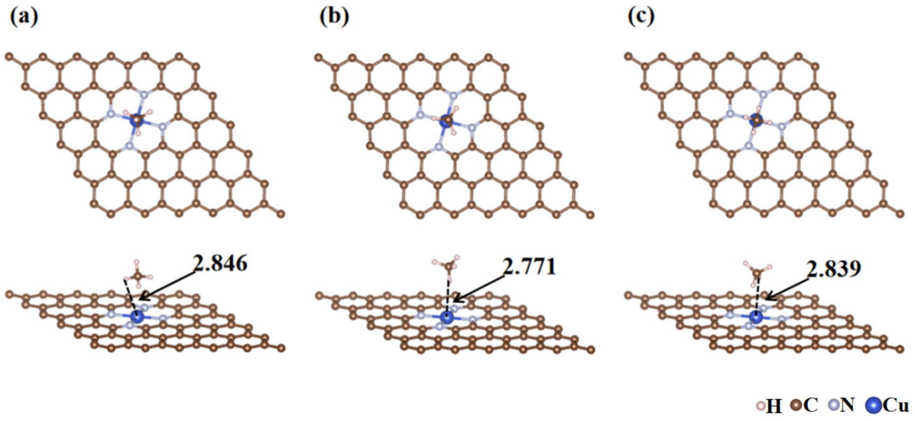

Figure S9. The structures of CH_4 on CuN_4-GPs: (a) $(CH_4)_{3H\text{-}down}$; (b) $(CH_4)_{3H\text{-}up}$; (c) $(CH_4)_{2H\text{-}down}$.

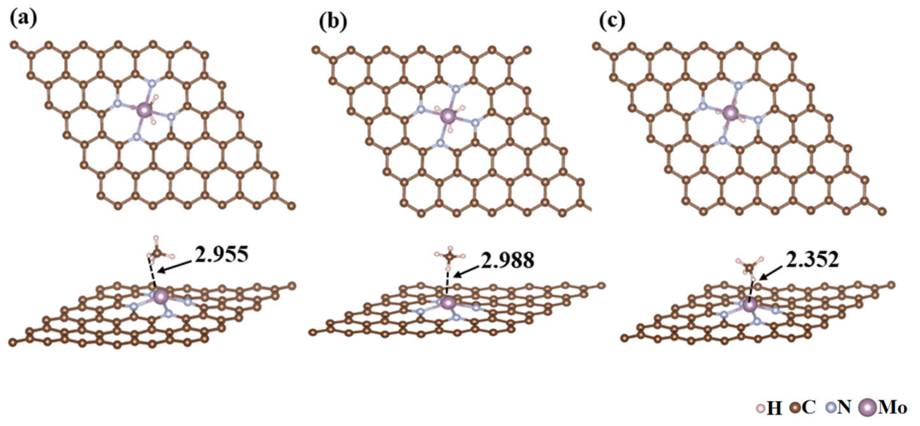

Figure S10. The structures of CH_4 on MoN_4-GPs: (a) $(CH_4)_{3H\text{-down}}$; (b) $(CH_4)_{3H\text{-up}}$; (c) $(CH_4)_{2H\text{-down}}$.

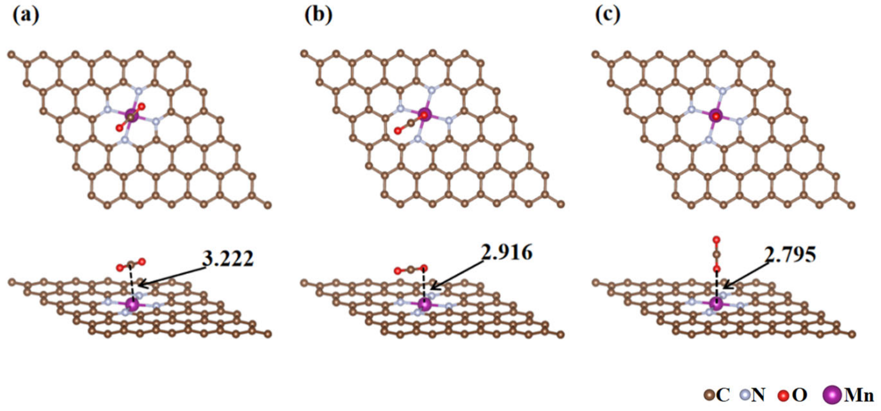

Figure S11. The structures of CO_2 on MnN_4-GPs: (a) $(CO_2)_{O\text{-paralle}}$; (b) $(CO_2)_{C\text{-parallel}}$; (c) $(CO_2)_{O\text{-vertical}}$.

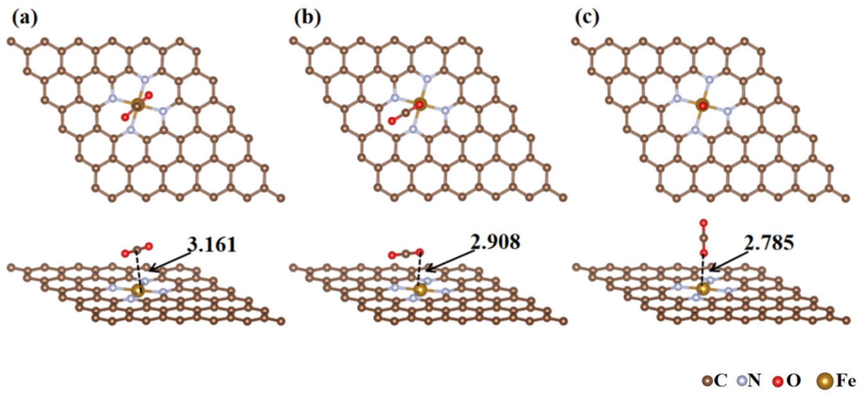

Figure S12. The structures of CO_2 on FeN_4-GPs: (a) $(CO_2)_{O\text{-paralle}}$; (b) $(CO_2)_{C\text{-parallel}}$; (c) $(CO_2)_{O\text{-vertical}}$.

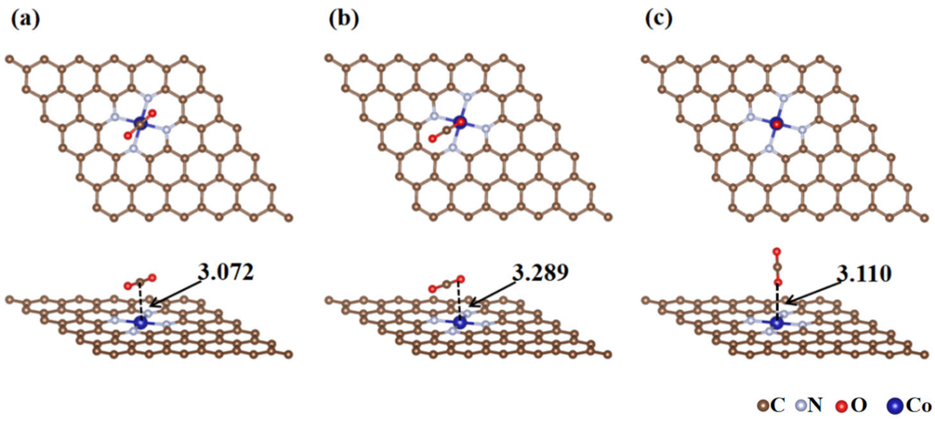

Figure S13. The structures of CO_2 on CoN_4-GPs: (a) $(CO_2)_{O\text{-paralle}}$; (b) $(CO_2)_{C\text{-parallel}}$; (c) $(CO_2)_{O\text{-vertical}}$.

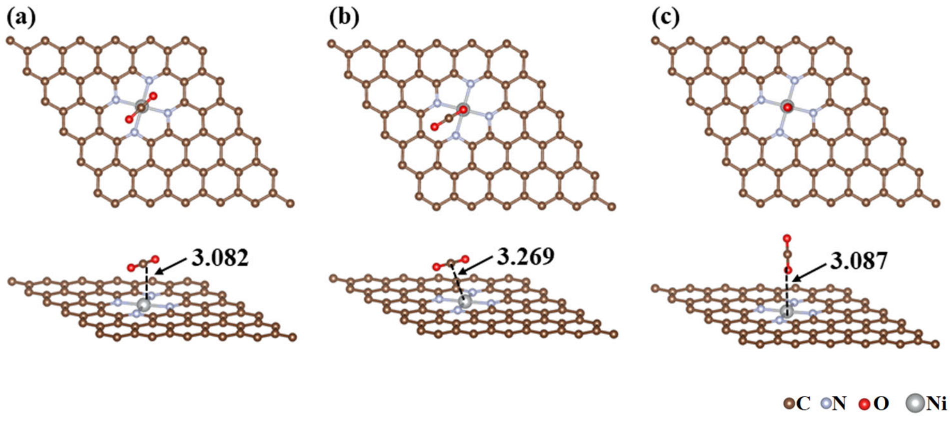

Figure S14. The structures of CO_2 on NiN_4-GPs: (a) $(CO_2)_{O\text{-paralle}}$; (b) $(CO_2)_{C\text{-parallel}}$; (c) $(CO_2)_{O\text{-vertical}}$.

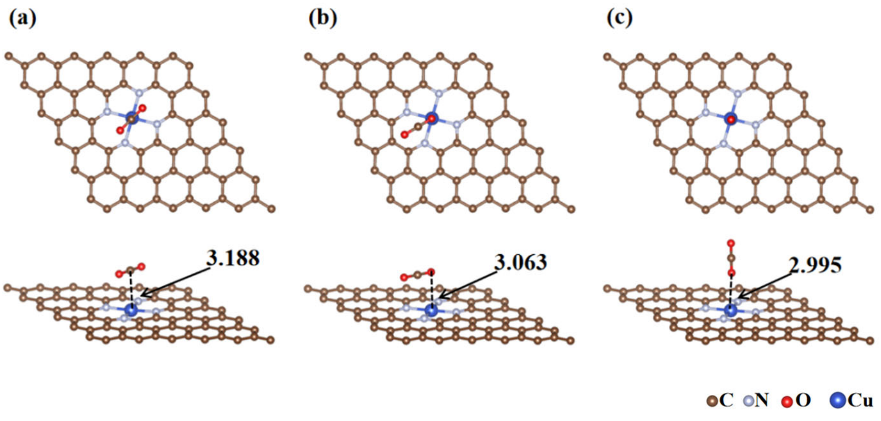

Figure S15. The structures of CO_2 on CuN_4-GPs: (a) $(CO_2)_{O\text{-paralle}}$; (b) $(CO_2)_{C\text{-parallel}}$; (c) $(CO_2)_{O\text{-vertical}}$.

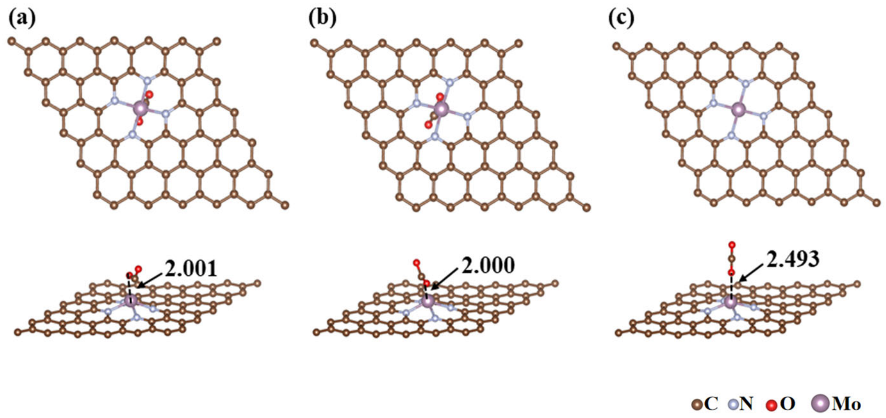

Figure S16. The structures of CO_2 on MoN_4-GPs: (a) $(CO_2)_{O\text{-paralle}}$; (b) $(CO_2)_{C\text{-parallel}}$; (c) $(CO_2)_{O\text{-vertical}}$.

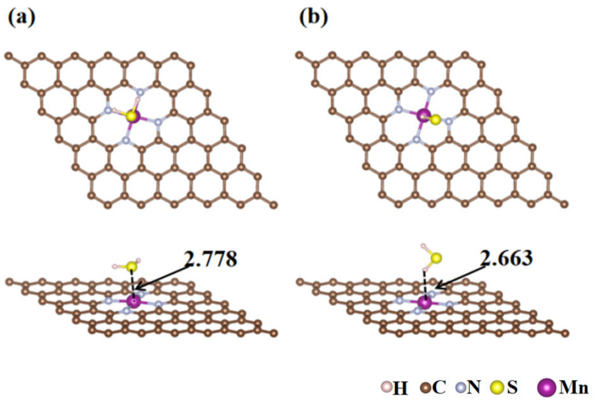

Figure S17. The structures of H_2S on MnN_4-GPs: (a) $(H_2S)_{parallel}$; (b) $(H_2S)_{vertical}$.

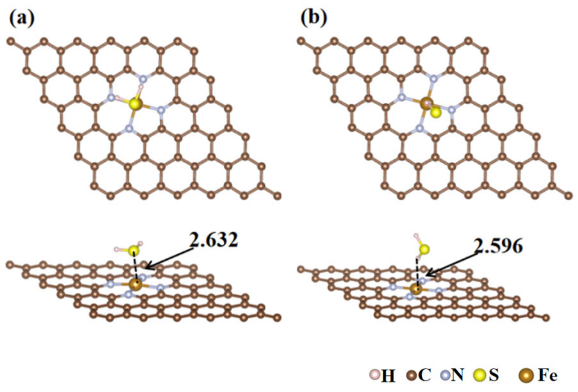

Figure S18. The structures of H_2S on FeN_4-GPs: (a) $(H_2S)_{parallel}$; (b) $(H_2S)_{vertical}$.

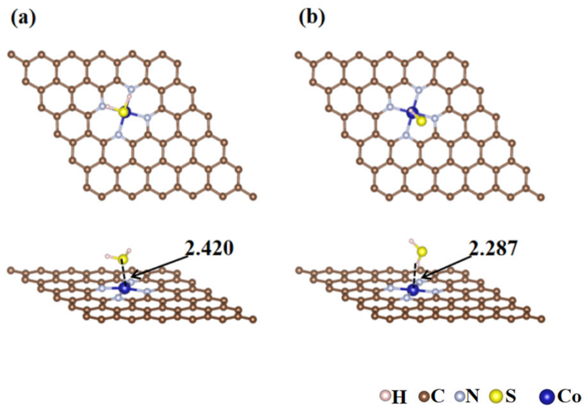

Figure S19. The structures of H_2S on CoN_4-GPs: (a) $(H_2S)_{parallel}$; (b) $(H_2S)_{vertical}$.

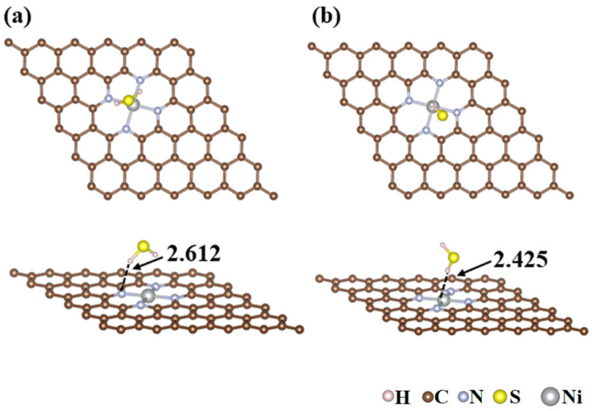

Figure S20. The structures of H_2S on NiN_4-GPs: (a) $(H_2S)_{parallel}$; (b) $(H_2S)_{vertical}$.

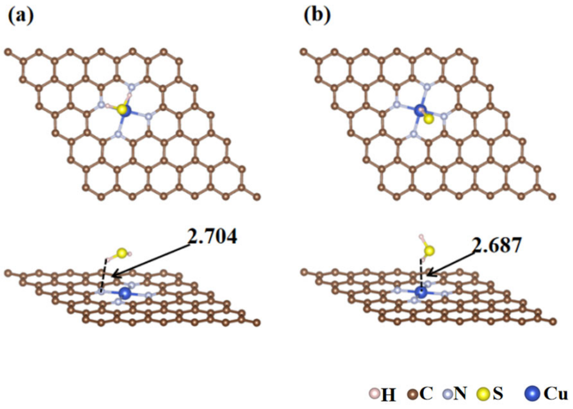

Figure S21. The structures of H_2S on CuN_4-GPs: (a) $(H_2S)_{parallel}$; (b) $(H_2S)_{vertical}$.

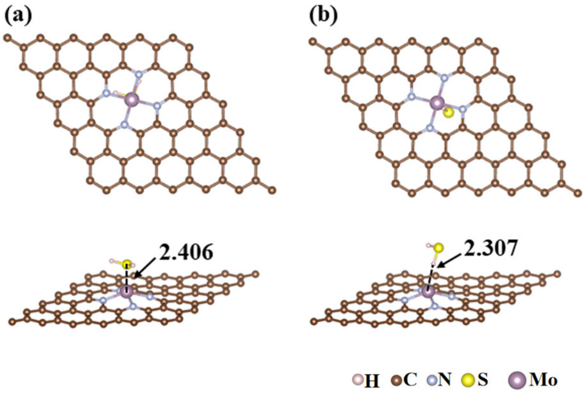

Figure S22. The structures of H_2S on MoN_4-GPs: (a) $(H_2S)_{parallel}$; (b) $(H_2S)_{vertical}$.

Table S1. The adsorption energies (eV) of CH_4, CO_2 and H_2S on MN_4-GPs (M = Mn, Fe, Co, Ni, Cu, Mo).

Molecules	MnN_4-GP	FeN_4-GP	CoN_4-GP	NiN_4-GP	CuN_4-GP	MoN_4-GP	Ref.
$(CH_4)_{3H\text{-}down}$	−0.189	−0.195	−0.117	−0.136	−0.159	−0.102	-
$(CH_4)_{3H\text{-}up}$	−0.112	−0.114	−0.115	−0.113	0.064	−0.084	-
$(CH_4)_{2H\text{-}down}$	−0.177	−0.180	−0.110	−0.131	0.023	−0.103	-
$(CO_2)_{C\text{-}parallel}$	−0.122	−0.130	−0.185	−0.168	0.029	−1.591	-
$(CO_2)_{O\text{-}parallel}$	−0.180	−0.184	−0.178	−0.165	−0.168	−1.587	-
$(CO_2)_{O\text{-}vertical}$	−0.133	−0.110	−0.044	−0.061	0.091	0.021	-
$(H_2S)_{S\text{-}parallel}$	−0.335	−0.380	−0.320	−0.060	−0.187	−0.897	-
$(H_2S)_{H\text{-}vertical}$	−0.125	−0.139	−0.241	−0.186	−0.136	−0.199	-
H_2S	-	−0.371	-	-	-	-	[1]
CO_2	-	−0.154	-	-	-	-	[1]
CO_2	-	−0.054	−0.212	−0.116	−0.280	-	[2]
CH_4	-	−0.157	−0.052	−0.109	−0.120	-	[2]

References

1. Nosheen, U.; Jalil, A.; Llyas, S.Z.; Ahmed, S.; Lllahi, A.; Rafiq, M.A. Ab-initio characterization of iron-embedded nitrogen-doped graphene as a toxic gas sensor. *J. Comput. Electron.* **2023**, *22*, 116–127.
2. Chen, S.Q.; Zhang, X.; Xiang, Y.C.; Fan, J.; Gan, L.Y. Computational screening of single transition metal atom embedded in nitrogen doped graphene for CH_4 detection. *Mater. Today Commun.* **2022**, *31*, 103383.
